# Phase Separation of PHLDB2 Drives EMT and Tumor Progression in Triple‐Negative Breast Cancer

**DOI:** 10.1002/cam4.71308

**Published:** 2025-11-30

**Authors:** Min Zhang, Chanjin Liang, Ting Chen, Xingyuan Shi, Jiqing Hao

**Affiliations:** ^1^ Department of Oncology The First Affiliated Hospital of Anhui Medical University Hefei China; ^2^ Department of Radiation Oncology The Fifth Hospital of Guangzhou Medical University Guangzhou China

**Keywords:** epithelial–mesenchymal transition, extracellular matrix, liquid–liquid phase separation, PHLDB2, triple‐negative breast cancer

## Abstract

**Background:**

Liquid–liquid phase separation (LLPS) has emerged as a critical mechanism underlying oncogenic signaling and transcriptional regulation; however, its involvement in triple‐negative breast cancer (TNBC) remains largely unknown. The pleckstrin homology‐like domain family B member 2 (PHLDB2) has been implicated in cytoskeletal organization and cell migration, but its role in phase separation–mediated tumor progression has not been explored.

**Methods:**

We conducted an integrative multi‐omics analysis combining transcriptomic profiling, bioinformatic modeling, and molecular biology experiments to elucidate the function of PHLDB2 in TNBC. Expression, mutation, and copy number variation of phase separation–related genes were analyzed using TCGA, METABRIC, and GTEx datasets. PHLDB2 knockdown and overexpression models were established in MDA‐MB‐231 and HCC38 cells to assess cell proliferation, epithelial–mesenchymal transition (EMT), and extracellular matrix (ECM)–related pathways. Immunofluorescence and fluorescence recovery after photobleaching (FRAP) assays were performed to evaluate the phase separation properties of PHLDB2. In vivo, xenograft and pulmonary metastasis models were used to validate the effects of Phldb2 knockdown on TNBC growth and metastasis.

**Results:**

PHLDB2 was significantly upregulated in TNBC and correlated with poor clinical prognosis. Gene ontology and KEGG analyses revealed its enrichment in ECM–receptor interaction and focal adhesion pathways. PHLDB2 knockdown inhibited cell proliferation and migration, suppressed EMT by upregulating E‐cadherin and downregulating N‐cadherin, vimentin, Snail, and MMP‐2. PONDR analysis identified extensive intrinsically disordered regions (IDRs) within PHLDB2, and FRAP assays confirmed its ability to form dynamic, reversible condensates consistent with LLPS. In vivo, Phldb2 depletion markedly reduced tumor growth and pulmonary metastases and prolonged survival in TNBC‐bearing mice.

**Conclusion:**

Our findings demonstrate that PHLDB2 promotes TNBC progression by mediating ECM remodeling and EMT activation through phase separation–driven signal organization. These results establish PHLDB2 as a novel phase separation–dependent oncogenic regulator and highlight its potential as a prognostic biomarker and therapeutic target in TNBC.

## 1 Introduction

Triple‐negative breast cancer (TNBC) is a highly heterogeneous disease known for its aggressive characteristics. The absence of estrogen receptors (ER), progesterone receptors (PR), and human epidermal growth factor receptor 2 (HER2) limits the effectiveness of conventional endocrine therapies and chemotherapy [1, 2]. Additionally, the invasive diversity of these tumors complicates treatment strategies, highlighting the urgent need for new therapeutic approaches targeting TNBC.

LLPS in biomolecules primarily arises from multivalent weak interactions that can quickly form, dissociate, and recombine. Proteins utilize tandem binding modules to achieve multivalency, often incorporating intrinsically disordered regions (IDRs) [3, 4, 5, 6, 7, 8]. Recent research has identified abnormal forms of liquid–liquid phase separation (LLPS) as significant factors influencing various cancer traits. Understanding the mechanisms behind abnormal LLPS condensates in cancer could provide valuable insights into how chromatin structure regulates gene expression. A notable example is the LLPS between nucleoporin 98 (NUP98) of the nuclear pore complex (NPC) and homeobox protein A9 (HOXA9), which contributes to the formation of extensive super‐enhancer (SE)‐like binding patterns, thereby promoting the transcriptional activation of leukemia‐causing genes [9]. Additionally, our previous research demonstrated that LLPS formed by HOXB8 and Fos‐related antigen 1 at chromatin SE sites establishes dysregulated transcription in osteosarcoma [10]. In triple‐negative breast cancer (TNBC) cells, phosphorylated histone deacetylase 6 (phospho‐HDAC6) forms LLPS condensates within the nucleus, which are essential for establishing abnormal chromatin structures [11]. Despite these exciting discoveries, efforts to elucidate the molecular functions of LLPS condensates in breast cancer, particularly in TNBC, remain in their early stages.

PHLDB2, a member of the PHLDB family, has garnered relatively little attention in cancer research [12, 13, 14, 15]. Recent studies have suggested its involvement in several cancers, including colorectal, lung, rectal, gastric, renal, glioma, and head and neck squamous cell carcinoma [16, 17, 18, 19, 20, 21, 22]. However, PHLDB2's role in TNBC has yet to be investigated. Initially recognized as LL5β, PHLDB2 features a PH domain essential for binding phosphoinositide phosphates and activating EGFR signaling. While the function of its PH domain is somewhat understood, the roles of other amino acid regions require further exploration, particularly concerning PHLDB2's involvement in abnormal LLPS in breast cancer.

In this study, we examined the role of phase separation‐related genes (PRGs) in TNBC. Our findings revealed significant changes in the expression, mutations, and copy number variations of 22 PSRGs in TNBC patients. By developing a prognostic model, we identified PRGs, especially PHLDB2, as potential prognostic indicators. Furthermore, we demonstrated the strong phase separation characteristics of PHLDB2 and its critical role in TNBC progression and tumorigenesis. Mechanistically, PHLDB2 deficiency was found to inhibit epithelial‐mesenchymal transition (EMT) at both the RNA level and the protein level. Overall, our results highlight the importance of PHLDB2 in TNBC, positioning it as a promising prognostic marker and therapeutic target.

## 2 Result

### 2.1 Differential Expression of PHLDB2 and Its Prognostic Significance in Triple Negative Breast Cancer (TNBC)

**FIGURE 1:**
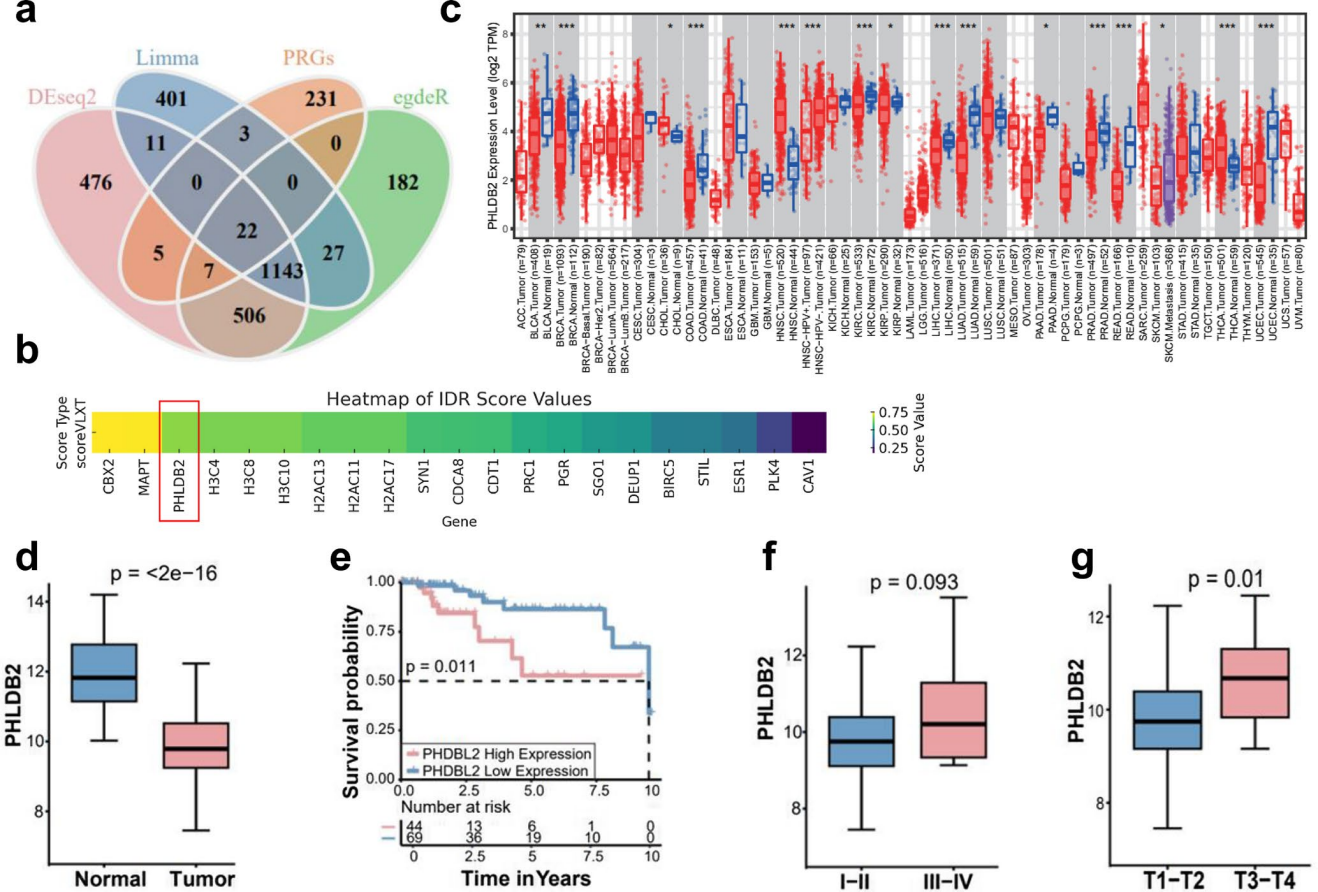
Expression pattern and clinical relevance of PHLDB2 in pan‐cancer analysis. (a) Venn diagram showing the intersection of differentially expressed genes (DEGs) identified by four methods: DESeq2 (pink), Limma (blue), edgeR (green), and PRGs (orange). The overlapping section represents commonly identified DEGs. (b) Heatmap of intrinsically disordered region (IDR) scores for the 22 intersected genes. PHLDB2 showed one of the highest IDR scores, suggesting strong phase separation potential. (c) Boxplot comparing PHLDB2 expression levels between tumor (red) and normal (blue) tissues across multiple cancer types. Statistical significance is indicated (*p < 0.05, **p < 0.01, ***p < 0.001). (d) Boxplot showing PHLDB2 expression in normal versus tumor tissues. Statistical significance is denoted by *p* < 2e−16. (e) Kaplan–Meier survival curve comparing overall survival probability between patients with high (pink) and low (blue) PHLDB2 expression. The log‐rank test *p*‐value is 0.011. (f) Boxplot of PHLDB2 expression levels in early‐stage (I–II) versus late‐stage (III–IV) cancer patients. The *p*‐value is 0.093. (g) Boxplot of PHLDB2 expression levels in lower‐stage (T1–T2) versus higher‐stage (T3–T4) cancer patients, with a significant *p*‐value of 0.01.

In this study, we systematically analyzed the expression of PHLDB2 across multiple cancer types and its association with survival outcomes in triple negative breast cancer (TNBC) patients. Using three differential expression analysis methods (DEseq2, Limma, and edgeR), we identified three sets of differentially expressed genes. These gene sets were then intersected with phase separation‐related genes (PRGs), resulting in 22 common genes: BIRC5, SGO1, H2AC13, CDCA8, H2AC17, H3C8, PRC1, CBX2, CDT1, STIL, H3C4, PLK4, DEUP1, H3C10, H2AC11, SYN1, MAPT, PHLDB2, ESR1, PGR, CAV1 (Figure 1a). To further prioritize candidate genes with potential phase separation properties, we evaluated the intrinsically disordered region (IDR) scores of the 22 intersected genes. As shown in the heatmap (Figure 1b), PHLDB2 exhibited one of the highest IDR scores among all candidates, indicating a strong potential for intrinsic disorder and phase separation capacity. This suggests that PHLDB2 may function through liquid–liquid phase separation mechanisms, contributing to its role in tumor progression and cellular organization. These findings supported the selection of PHLDB2 as the core gene for subsequent analysis in TNBC. We analyzed PHLDB2 expression across various cancer types and found significantly lower expression in tumor tissues compared to normal tissues in most cancers (Figure 1c). This trend was also evident in TNBC, where PHLDB2 exhibited notably lower expression in tumor tissues compared to normal tissues (Figure 1d, *p* < 2e−16). Survival analysis demonstrated that high PHLDB2 expression was significantly associated with worse overall survival in TNBC patients (Figure 1e, *p* = 0.011). Furthermore, clinical staging analysis indicated a potential correlation between PHLDB2 expression and disease progression, with statistical differences between stages I‐II and III‐IV (Figure 1f, *p* = 0.093) and a significant difference between stages T1–T2 and T3–T4 (Figure 1g, *p* = 0.01).

### 2.2 Correlation of PHLDB2 With Biological Pathways and Immune Infiltration in TNBC

**FIGURE 2:**
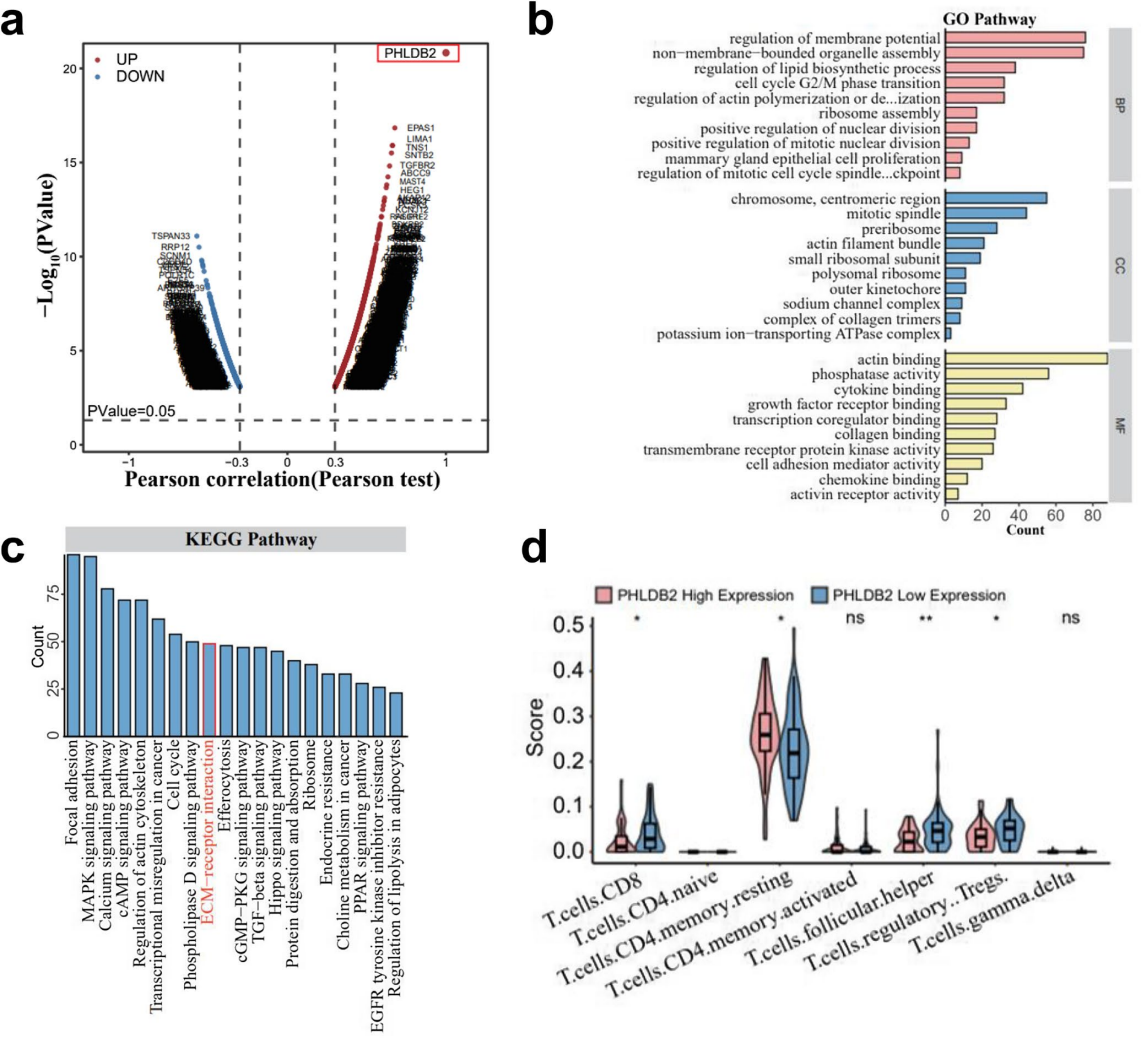
Functional enrichment and immune cell infiltration analysis related to PHLDB2 expression. (a) Scatter plot showing the Pearson correlation between PHLDB2 expression and other genes. Genes with a significant positive correlation (red) and negative correlation (blue) are highlighted. The horizontal dashed line represents the *p*‐value threshold (*p* = 0.05). PHLDB2 is circled in red. (b) Gene Ontology (GO) enrichment analysis for genes correlated with PHLDB2. GO terms are categorized into three groups: Biological Process (BP, pink), Cellular Component (CC, blue), and Molecular Function (MF, yellow). The *x*‐axis represents the gene count in each category. (c) Kyoto Encyclopedia of Genes and Genomes (KEGG) pathway enrichment analysis of PHLDB2‐correlated genes. The bar graph represents the number of genes enriched in each pathway. (d) Violin plots comparing immune cell infiltration scores between high and low PHLDB2 expression groups. Immune cell types are shown on the *x*‐axis, and infiltration scores are on the *y*‐axis. Statistical significance is indicated (*p < 0.05, **p < 0.01, ns, not significant).

We conducted a Pearson correlation analysis to explore the association between PHLDB2 expression and other genes in TNBC. The results revealed several genes significantly correlated with PHLDB2 expression (Figure 2a). Gene ontology (GO) enrichment analysis of PHLDB2‐associated genes highlighted their involvement in various biological processes (BP), cellular components (CC), and molecular functions (MF). These included regulation of membrane potential, non–membrane‐bounded organelle assembly, regulation of lipid biosynthetic process, and cell cycle G2/M phase transition (Figure 2b). KEGG pathway analysis further identified key pathways enriched in PHLDB2‐related genes, such as MAPK signaling pathway, focal adhesion, ECM–receptor interaction, and transcriptional misregulation in cancer (Figure 2c). Immune infiltration analysis showed that PHLDB2 expression was significantly associated with various immune cell types. Specifically, low PHLDB2 expression was correlated with increased infiltration of CD8+ T cells and Tregs, while high PHLDB2 expression was associated with resting CD4 memory (Figure 2d).

### 2.3 PHLDB2 Plays a Critical Role in TNBC Progression and Tumorigenesis

**FIGURE 3:**
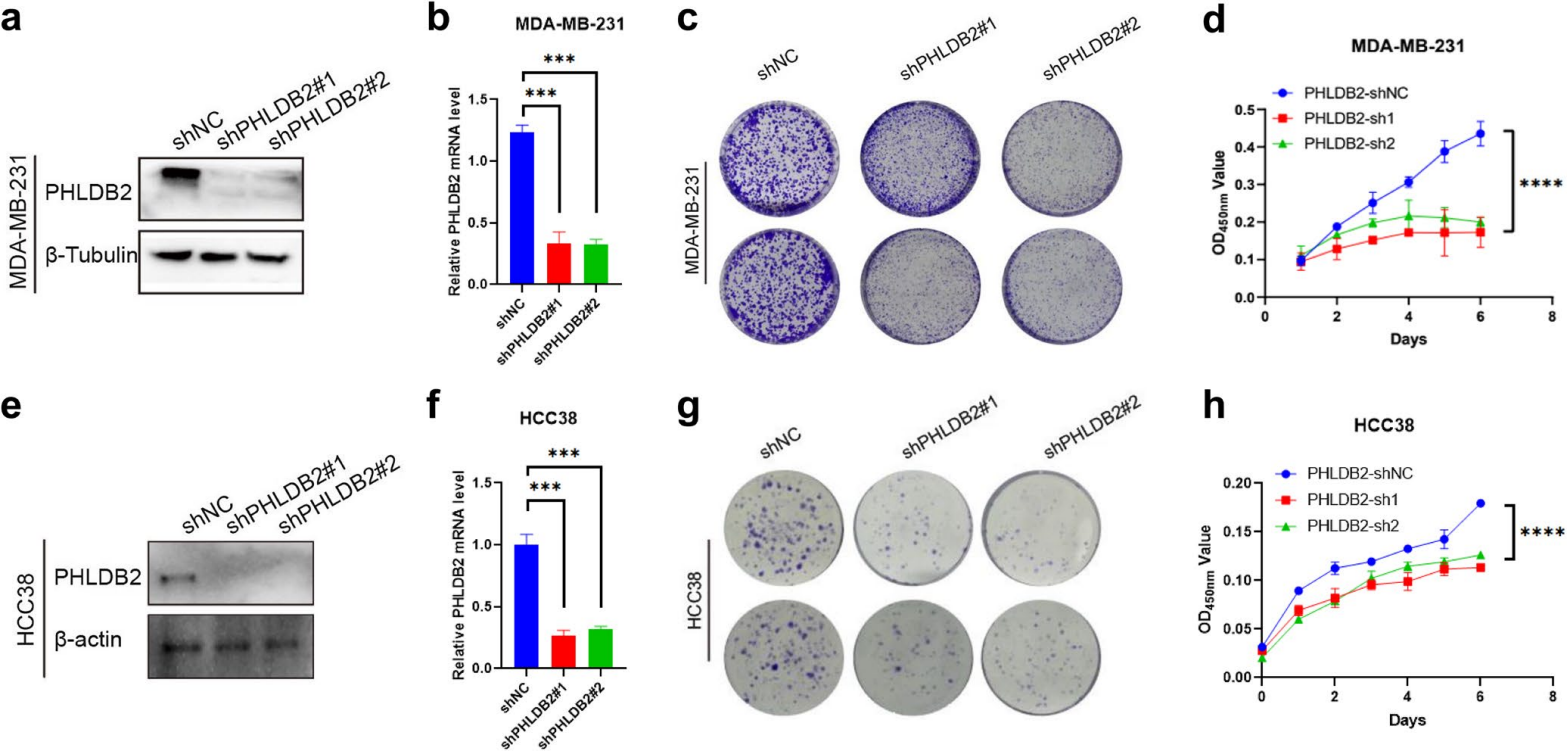
PHLDB2 knockdown inhibits proliferation of MDA‐MB‐231 and HCC38 breast cancer cells. (a) Western blot analysis of PHLDB2 protein expression in MDA‐MB‐231 cells transfected with non‐targeting control shRNA (shNC) or two independent PHLDB2‐targeting shRNAs (shPHLDB2#1 and shPHLDB2#2). β‐Tubulin serves as a loading control. (b) Quantitative real‐time PCR analysis of PHLDB2 mRNA expression levels after knockdown in MDA‐MB‐231 cells ***p < 0.001. (c) Representative images of colony formation assays in MDA‐MB‐231 cells with PHLDB2 knockdown (shPHLDB2#1 and shPHLDB2#2) and control (shNC). (d) Cell proliferation assay (OD450 nm) showing the growth of MDA‐MB‐231 cells over time with PHLDB2 knockdown (shPHLDB2#1 and shPHLDB2#2) compared to control (shNC) ****p < 0.0001. (e) Western blot analysis of PHLDB2 expression in HCC38 cells transfected with shNC, shPHLDB2#1, or shPHLDB2#2. β‐Tubulin is used as a loading control. (f) Quantitative real‐time PCR analysis of PHLDB2 mRNA expression levels after knockdown in HCC38 cells ***p < 0.001. (g) Representative images of colony formation assays in HCC38 cells with PHLDB2 knockdown (shPHLDB2#1 and shPHLDB2#2) and control (shNC). (h) Cell proliferation assay (OD450 nm) showing the growth of HCC38 cells over time with PHLDB2 knockdown (shPHLDB2#1 and shPHLDB2#2) compared to control (shNC) ****p < 0.0001.

To assess the effect of PHLDB2 on TNBC progression, we used two different short hairpin RNAs (shRNAs) to stably knock down PHLDB2 expression in MDA‐MB‐231 and HCC38 cells. Western blot and qRT‐PCR analyses confirmed efficient knockdown of PHLDB2 at both the protein and mRNA levels in MDA‐MB‐231 (Figure 3a,b) and HCC38 cells (Figure 3e,f). The colony formation assay confirmed a significant decrease in the frequency of colony formation in shPHLDB2 cells, indicating that the colony formation ability of TNBC cells was weakened after the reduction of PHLDB2 (Figure 3c,g). Using the CCK‐8 detection method, we observed a decrease in the growth rate of MDA‐MB‐231 and HCC38 cells with reduced PHLDB2 expression compared to control cells (Figure 3d,h). Together, these findings emphasize the critical role of PHLDB2 in promoting TNBC cell proliferation in vitro. These results underscore the critical role of PHLDB2 in TNBC progression and tumorigenesis.

### 2.4 PHLDB2 Knockdown in TNBC Alters ECM‐Receptor Interaction and EMT Pathways

**FIGURE 4:**
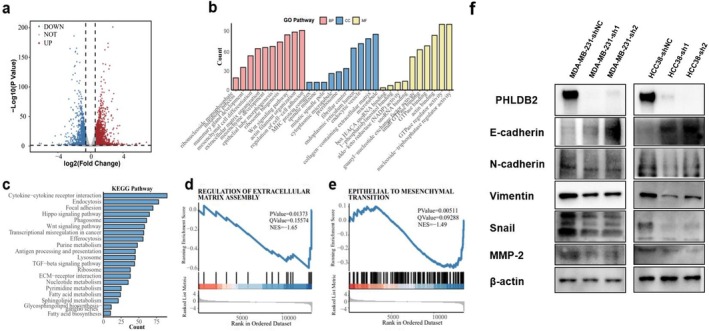
Transcriptomic and functional analysis reveals the role of PHLDB2 in epithelial–mesenchymal transition (EMT) and extracellular matrix regulation. (a) Volcano plot showing differentially expressed genes upon PHLDB2 knockdown. Upregulated genes (red) and downregulated genes (blue) are highlighted, while non‐significant genes are shown in gray. The *x*‐axis represents log2(Fold Change), and the *y*‐axis represents −log10(*p*‐value). (b) Gene Ontology (GO) enrichment analysis of differentially expressed genes, categorized into Biological Process (BP, pink), Cellular Component (CC, blue), and Molecular Function (MF, yellow). The *x*‐axis shows enriched GO terms, while the *y*‐axis represents the gene count. (c) Kyoto Encyclopedia of Genes and Genomes (KEGG) pathway enrichment analysis of differentially expressed genes. The *x*‐axis represents the gene count, and the *y*‐axis lists the significantly enriched pathways. (d, e) Gene Set Enrichment Analysis (GSEA) plots showing the negative enrichment of the “Regulation of Extracellular Matrix Assembly” (d) and “Epithelial to Mesenchymal Transition” (e) pathways in PHLDB2 knockdown cells. The *x*‐axis represents the rank in the ordered dataset, and the *y*‐axis shows the running enrichment score. *p*‐values, *q*‐values, and normalized enrichment scores (NES) are indicated. (f) Western blot analysis of PHLDB2, E‐cadherin, N‐cadherin, Vimentin, Snail, and MMP‐2 expression in MDA‐MB‐231 and HCC38 cells with PHLDB2 knockdown (shPHLDB2#1 and shPHLDB2#2) compared to the control (shNC). β‐actin serves as a loading control.

We performed differential expression analysis between PHLDB2 knockdown (SH) and negative control (NC) samples in MDA‐MB‐231 cells (Figure 4a). Several genes were significantly upregulated (red) or downregulated (blue) after PHLDB2 knockdown. Gene Ontology (GO) enrichment analysis (Figure 4b) revealed involvement in key processes such as regulation of cell–cell adhesion, Wnt signaling pathway, extracellular matrix organization, and mesenchymal cell differentiation. KEGG pathway enrichment analysis (Figure 4c) revealed significant pathways such as cytokine‐cytokine receptor interaction, focal adhesion, and ECM–receptor interaction. Furthermore, GSEA analysis (Figure 4d,e) showed enrichment of epithelial‐mesenchymal transition (EMT) (*p*‐value = 0.00511, NES = −1.49) and extracellular matrix assembly (ECM) (*p*‐value = 0.01373, NES = −1.65), indicating a potential role for PHLDB2 in these processes following knockdown. Therefore, we investigated the effect of PHLDB2 knockdown on the EMT signaling pathway and showed that knockdown of PHLDB2 in MDA‐MB‐231 and HCC38 cells resulted in the up‐regulation of E‐cadherin proteins and the down‐regulation of N‐cadherin, vimentin, snail, and MMP‐2 proteins, which suggested that knockdown of PHLDB2 inhibited epithelial mesenchymal transition in TNBC (Figure 4f).

### 2.5 PHLDB2 Exhibits Robust Phase Separation Properties

**FIGURE 5:**
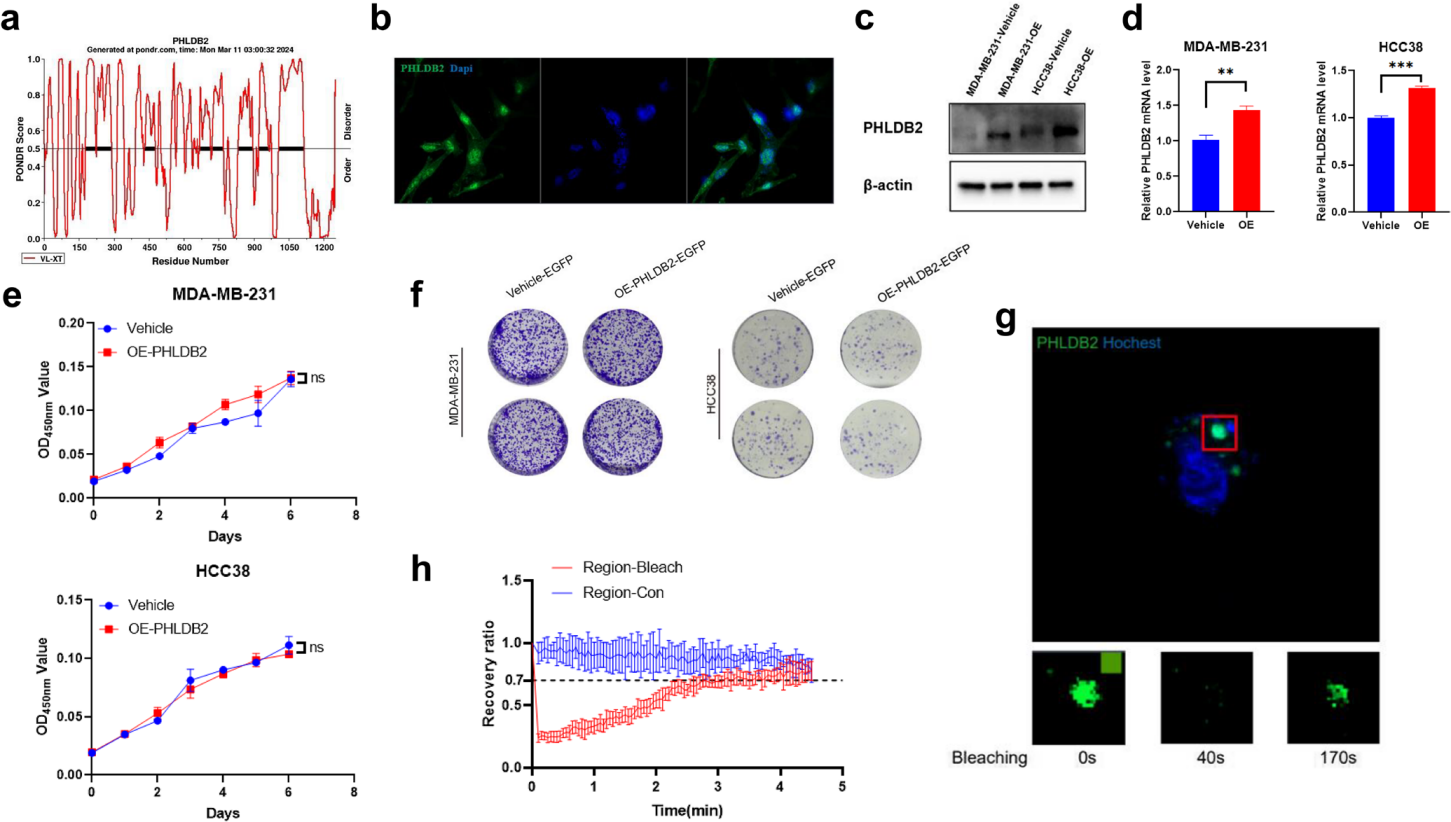
Structural prediction, subcellular localization, and functional effects of PHLDB2 overexpression in breast cancer cells. (a) PONDR analysis predicting the intrinsic disorder tendency of PHLDB2 protein. The *x*‐axis represents the residue number, while the *y*‐axis indicates the PONDR score. Scores above 0.5 suggest intrinsically disordered regions. (b) Immunofluorescence staining showing the localization of PHLDB2 (green) in cells. DAPI (blue) stains the nuclei. (c) Western blot analysis confirming the overexpression of PHLDB2 in MDA‐MB‐231 and HCC38 cell lines compared to vehicle control. β‐actin serves as a loading control. (d) Quantitative real‐time PCR analysis of PHLDB2 mRNA expression levels after overexpressed in MDA‐MB‐231 and HCC38 cells **p < 0.01, ***p < 0.001. (e) CCK‐8 assay showing the proliferation of MDA‐MB‐231 (top) and HCC38 (bottom) cells with PHLDB2 overexpression (OE‐PHLDB2) compared to the vehicle control. OD450 nm values are plotted over time. “ns” indicates no significant difference. (f) Colony formation assay of MDA‐MB‐231 (left) and HCC38 (right) cells with PHLDB2 overexpression (OE‐PHLDB2‐EGFP) compared to the vehicle control. (g) Fluorescence recovery after photobleaching (FRAP) experiment showing PHLDB2 dynamics in live cells. The top panel shows an image of a bleached region (red box), while the lower panels show fluorescence recovery at different time points (0s, 40s, 170s). (h) Quantification of FRAP results, showing the fluorescence recovery ratio over time. The red line represents the bleached region, and the blue line represents the control region. The *x*‐axis indicates time (minutes), and the *y*‐axis shows the recovery ratio.

In order to delve into the phase separation properties of PHLDB2, we first scrutinized its intrinsic disorder regions (IDRs) through the PONDR website. Eight highly disordered regions were found outside the Pleckstrin homology structural domain (1143–1246), spanning amino acid sites 178–1115, laying the structural foundation for the phase‐separation properties of PHLDB2 (Figure 5a). Subsequently, we assessed the expression of endogenous PHLDB2 protein in triple‐negative breast cancer cells by immunofluorescence, which revealed the presence of liquid‐like punctate protrusions (Figure 5b), suggesting a possible phase separation. To confirm the phase‐segregation property of PHLDB2 in vivo, we overexpressed EGFP‐PHLDB2 protein in the triple‐negative breast cancer cell line MDA‐MB‐231 (Figure 5c,d) and performed fluorescence recovery after photobleaching (FRAP) assays. The results showed that overexpression of PHLDB2 did not affect cell proliferation and clone formation (Figure 5e,f). The results of FRAP experiments showed that EGFP‐PHLDB2 condensate recovered dynamically after photobleaching and reached 60% of the pre‐bleaching intensity after about 170 s (Figure 5g,h), which further confirmed the phase‐separation behavior of PHLDB2 in the cellular environment.

### 2.6 Knockdown of PHLDB2 Significantly Suppresses the Growth and Metastasis of TNBC In Vivo

**FIGURE 6:**
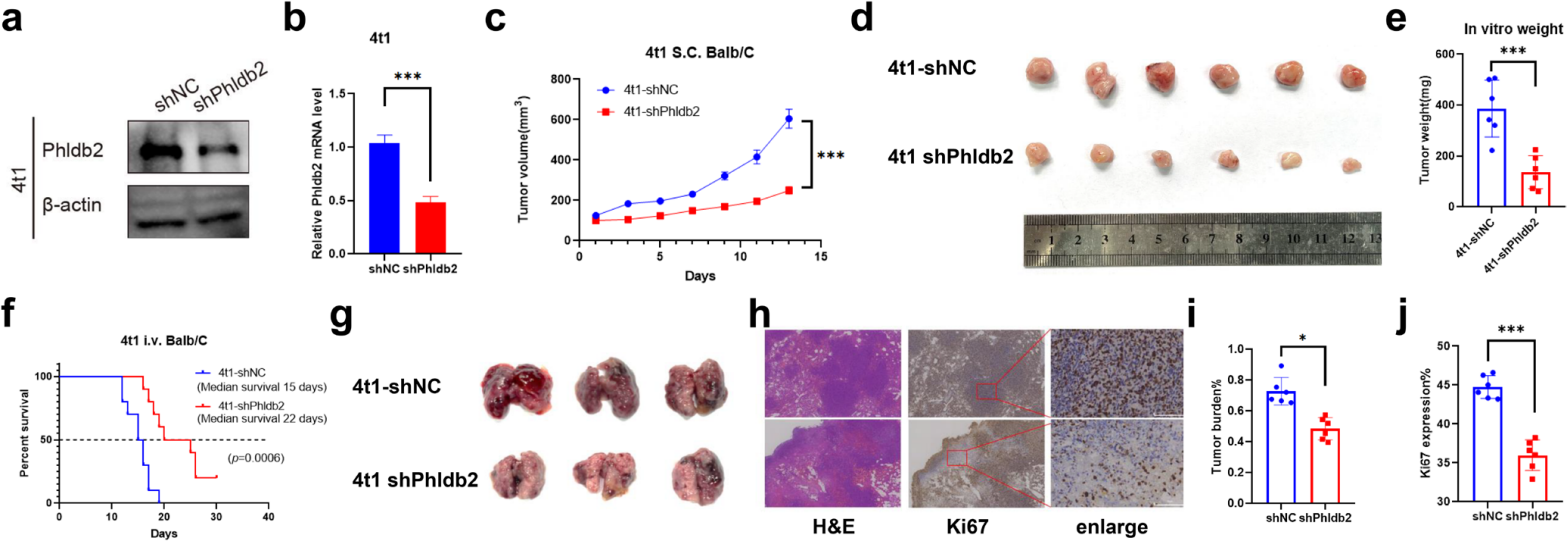
Knockdown of Phldb2 suppresses tumor growth and metastasis in 4T1 breast cancer models. (a) Western blot analysis of Phldb2 expression in 4T1 cells transfected with shNC (negative control) or shPhldb2. β‐Tubulin serves as a loading control. (b) Quantitative real‐time PCR analysis of PHLDB2 mRNA expression levels after knockdown in 4t1 cells. (c) Tumor growth curve showing the tumor volume of 4t1‐shNC and 4t1‐shPhldb2 groups over time in Balb/C mice. Tumor volume was significantly reduced in the 4t1‐shPhldb2 group compared to the control group (*n* = 6, ***p < 0.001). (d) Representative images of excised tumors from 4t1‐shNC and 4t1‐shPhldb2 groups. (e) Quantification of tumor weight from the excised tumors in panel (d). The tumor weight was significantly lower in the 4t1‐shPhldb2 group compared to the control (***p < 0.001). (f) Kaplan–Meier survival curve for Balb/C mice injected intravenously (i.v.) with 4t1‐shNC or 4t1‐shPhldb2 cells. The median survival was significantly longer in the 4t1‐shPhldb2 group (22 days) compared to the 4t1‐shNC group (15 days) (*n* = 10, *p* = 0.0006). (g) Representative images of lung metastases in mice injected intravenously with 4t1‐shNC or 4t1‐shPhldb2 cells. The 4t1‐shPhldb2 group showed fewer metastatic nodules compared to the 4t1‐shNC group. (h) Histological analysis of lung metastases by H&E staining and immunohistochemistry for Ki67. Enlarged views show Ki67‐positive cells. (i) Quantification of lung tumor burden as a percentage of lung area occupied by tumors (*n* = 6, *p < 0.05). (j) Quantification of Ki67 expression in lung metastatic lesions (*n* = 6, ***p < 0.001).

To further investigate the role of PHLDB2 in tumor progression in vivo, we established a stable Phldb2‐knockdown 4t1 cell line (4t1‐shPhldb2) using the murine TNBC cell line 4t1 (Figure 6a,b). We then generated a subcutaneous xenograft model and a pulmonary metastasis model of TNBC in mice to evaluate the in vivo effects of Phldb2. Analysis of tumor growth curves revealed that the Phldb2‐knockdown group exhibited significantly slower tumor progression (Figure 5c–e). In the TNBC lung metastasis model, mice with Phldb2 knockdown showed prolonged survival (Figure 5f) and a marked reduction in pulmonary tumor burden (Figure 5g–j). These findings demonstrate that Phldb2 knockdown effectively suppresses TNBC growth and significantly extends survival in mice with TNBC lung metastasis.

## 3 Discussion

Emerging evidence implicates phase separation‐associated gene sets as novel oncogenic drivers across multiple cancer types. Our study systematically extends this paradigm to triple‐negative breast cancer (TNBC), establishing their pathological relevance in this aggressive malignancy. Methodologically, we employed an integrated bioinformatics approach combining three distinct differential expression analysis pipelines (DESeq2, limma‐voom, and edgeR) to comprehensively characterize liquid–liquid phase separation (LLPS)‐related transcriptional alterations in TNBC. This multi‐algorithm consensus analysis identified 22 high‐confidence LLPS‐associated genes exhibiting significant dysregulation. Of particular translational importance, we mechanistically demonstrated the pivotal involvement of PHLDB2 in TNBC progression through functional validation studies. These findings not only expand the oncogenic repertoire of phase separation regulators but also provide a molecular framework for investigating LLPS‐mediated pathogenesis in TNBC.

PHLDB2 (also known as LL5β) is a PRICKLE1‐associated adaptor protein [23]. As a pleckstrin homology (PH) domain‐containing protein, PHLDB2 exhibits partial membrane localization [24] and functions to anchor microtubules (MTs) through direct interaction with cytoplasmic linker‐associated proteins (CLASPs), members of the MT plus‐end tracking protein family [25]. The PHLDB2‐CLASP interaction facilitates focal adhesion disassembly and promotes cell migration [26], serving as an independent prognostic marker for poor tumor outcomes. While PHLDB2 has been conventionally implicated in cancer prognosis through its role in cell migration, its potential capacity for phase separation remains unreported to date.

Our study elucidates novel functional insights into PHLDB2 in triple‐negative breast cancer (TNBC). Genetic ablation of PHLDB2 induced significant alterations in RNA expression profiles, with knockdown experiments demonstrating downregulation of epithelial‐mesenchymal transition (EMT)–related pathways. These transcriptional changes were corroborated by corresponding protein‐level modifications in western blot analyses, suggesting PHLDB2's regulatory role in tumor cell proliferation and migration through EMT modulation.

Notably, we made the seminal observation that intracellular PHLDB2 can form phase‐separated condensates, implying a potential mechanistic link between its phase separation properties and transcriptional regulatory functions. Given that PHLDB proteins contain a phosphoinositide‐binding pleckstrin homology (PH) domain, our findings warrant further investigation into whether PHLDB2 mediates phospholipid incorporation into its phase‐separated assemblies through distinct structural domains, or modulates phosphoinositide‐associated signaling pathways via phase separation mechanisms, both of which may contribute to TNBC progression.

In summary, our study establishes a novel prognostic model based on phase separation‐associated genes in TNBC. We have demonstrated the critical role of PHLDB2 in TNBC progression through its unique capacity for biomolecular phase separation and its regulatory effects on EMT‐related protein expression at both transcriptional and translational levels. These findings provide compelling evidence that PHLDB2 orchestrates phase separation‐mediated transcriptional reprogramming in TNBC, revealing its multifaceted functions in cancer pathogenesis. The mechanistic insights gained from this study open new avenues for developing targeted therapeutic strategies against this aggressive breast cancer subtype.

## 4 Methods

### 4.1 Data Acquisition and Processing

Expression data and clinical information for breast cancer (BRCA) were downloaded from the UCSC Xena database (https://xenabrowser.net/datapages/). Phase separation‐related genes (PRGs) were downloaded from the PhaSepDB database (http://db.phasep.pro). The expression data were normalized using the DEseq2 package in R. From the BRCA datasets, we extracted expression and clinical data specific to triple‐negative breast cancer (TNBC) for subsequent analyses. Additionally, we used the TIMER2.0 website (http://timer.cistrome.org) to analyze PHLDB2 expression across various cancer types to explore its differential expression in tumor versus normal tissues.

### 4.2 Differential Gene Expression Analysis and Enrichment Analysis

Differential expression analysis was performed using the DESeq2, edgeR, and limma (voom) packages in R. Genes with *p*‐value < 0.01, *q*‐value < 0.01, and absolute log fold change (logFC) > 1.5 were considered significantly differentially expressed. To visualize the overlap of differentially expressed genes between these methods, a Venn diagram was created using the VennDiagram package in R. GO enrichment analysis, KEGG pathway enrichment analysis and Gene Set Enrichment Analysis (GSEA) were performed using the “clusterProfiler” package in R.

### 4.3 Immune Infiltration Analysis

To assess the relationship between PHLDB2 expression and immune infiltration, we used the CIBERSORT algorithms. The infiltration scores of different immune cell subtypes were compared between high and low PHLDB2 expression groups, and significance was assessed using Wilcoxon rank‐sum tests.

### 4.4 Statistical Analysis

Comparisons of PHLDB2 expression between cancer and normal tissues, as well as between stages T1–T2 and T3–T4, and stages I–II and III–IV, were conducted using the Wilcoxon rank‐sum test. Survival analysis was performed using Kaplan–Meier survival curves, and the significance of differences between survival groups was assessed using the log‐rank test. Pearson correlation analysis was performed to identify genes significantly associated with PHLDB2 expression in TNBC. Correlation coefficients and *p*‐values were calculated, and genes with an absolute correlation coefficient > 0.3 and a *p*‐value < 0.05 were considered significant.

### 4.5 Cell Lines and Cell Culture

The human triple‐negative breast cancer cell line (MDA‐MB‐231 and HCC38) and the mice triple‐negative breast cancer cell line (4t1) were obtained from the Cell Bank of Shanghai Institutes for Biological Sciences (SIBS). These cell lines are cultured in Dulbecco's Modified Eagle Medium (DMEM, Gibco, USA) supplemented with 10% fetal bovine serum (FBS, Hyclone, Germany). The cultures are maintained in a humidified incubator at 37°C with 5% CO_2_.

### 4.6 Animal Experiments

8‐week‐old Balb/c female mice were maintained at specific‐pathogen‐free (SPF) health status in individually ventilated cages at 21°C–22°C and 39%–50% humidity, under 12 h light–dark cycles. The 4t1 cells were injected subcutaneously into the flanks of the mice or intravenously into the mice (1 × 10^6^ cells suspended in 100 μL sterile PBS). Measurements were performed every 2 days each after 7 days of injection. Tumor size in animal experiments did not exceed the limit of 2000 mm^3^. Tumor volume was measured regularly using vernier calipers and calculated using the formula [*V* = ½ (Length × Width^2^)].

### 4.7 Viral Preparation and Transduction

Using Lipofectamine 3000 (Invitrogen, USA), transfect LV‐shScramble, shPHLDB2#1, and shPHLDB2#2 vectors into 293T cells, along with the lentivirus packaging plasmids psPAX2 and pMD2G. After incubating for 72 h, collect and concentrate the supernatant containing the lentivirus. For transduction, seed the cells in a culture dish and expose them to the lentivirus and 10 μg/mL polybrene (Santa Cruz, USA) for 8 h.

### 4.8 Western Blot

The cell lysates were separated using 10% SDS‐PAGE and transferred to a PVDF membrane (Millipore, USA). The membrane was probed overnight at 4°C with primary antibodies, including PHLDB2 (Abcam, ab234885), E‐cadherin (PTC, 60335‐1‐Ig), N‐cadherin (PTC, 66219‐1‐Ig), Vimentin (PTC, 80232‐1‐RR), Snail (PTC, 13099‐1‐AP), MMP‐2 (PTC, 66366‐1‐Ig), and β‐actin (CST, #4967). Subsequently, the membrane was incubated at room temperature for 1 h with HRP‐conjugated goat anti‐rabbit IgG or HRP‐conjugated goat anti‐mouse IgG. Protein bands were visualized using a chemiluminescent imaging system (Bio‐Rad, USA), with β‐actin or GAPDH used as internal controls.

### 4.9 Immunofluorescence (IF)

IF staining was performed as previously described, using rabbit anti‐PHLDB2 antibody (Abcam, ab234885). The slides were counterstained with DAPI (Sigma) to visualize the cell nuclei and examined under a fluorescence microscope (Nikon, Japan).

### 4.10 Fluorescence Recovery After Photobleaching (FRAP) Assay

MDA‐MB‐231 cells were seeded onto 35‐mm confocal glass‐bottom dishes (NEST, China) and allowed to grow for 24 h. Then, transfect the cells with 2 μg of plasmid and culture for an additional 10 h. After a total transfection period of 72 h, conduct FRAP measurements using a 488 nm laser pulse focused on a single aggregate or region of interest (ROI). Measure the fluorescence intensity at 0.5‐s intervals, and then normalize it to the intensity recorded before bleaching.

### 4.11 Recombinant Protein Purification

To express the recombinant full‐length PHLDB2 protein, the pCDH vector containing the EGFP tag, Twin‐Strep tag, and full‐length PHLDB2 coding sequence was transfected into 293T cells. The 293T cells were cultured overnight at 37°C, and then PEI and plasmid were added at a final concentration for 16 h at 37°C. After 72 h of transfection, cells were harvested and lysed on ice in lysis buffer for 30 min. The lysate was sonicated and centrifuged at 13,000× *g* for 10 min. The supernatant was passed through a Gravity flow Strep‐TactinXT 4Flow column (IBA, 2‐5012‐001) and washed with washing buffer. The Strep‐tagged fusion protein was then eluted with elution buffer. The eluted protein was mixed with 5× SDS sample buffer, boiled, and analyzed by SDS‐PAGE. The protein was dialyzed against a urea solution from 8 to 0 M, concentrated to 1 M final concentration in 25 mM Tris–HCl pH 7.5, and stored at −80°C.

## Author Contributions

**Min Zhang:** validation, conceptualization, methodology, funding acquisition. **Chanjin Liang:** data curation, formal analysis. **Ting Chen:** data curation, formal analysis. **Xingyuan Shi:** supervision, writing – original draft. **Jiqing Hao:** writing – original draft Supervision.

## Ethics Statement

All animal experimental procedures were approved by the Animal Experiment Center of Anhui Medical University (20231253).

## Consent

The authors have nothing to report.

## Conflicts of Interest

The authors declare no conflicts of interest.

## Supporting information


**Data S1:** cam471308‐sup‐0001‐Figures.docx.

## Data Availability

The data that support the findings of this study are available from the corresponding author upon reasonable request.

## References

[1] G. Bianchini , J. M. Balko , I. A. Mayer , M. E. Sanders , and L. Gianni , “Triple‐Negative Breast Cancer: Challenges and Opportunities of a Heterogeneous Disease,” Nature Reviews. Clinical Oncology 13, no. 11 (2016): 674–690, 10.1038/nrclinonc.2016.66. PMC5461122 27184417

[2] Z. Wang , Q. Jiang , and C. Dong , “Metabolic Reprogramming in Triple‐Negative Breast Cancer,” Cancer Biology & Medicine 17, no. 1 (2020): 44–59, 10.20892/j.issn.2095-3941.2019.0210. 32296576 PMC7142847

[3] B. A. Gibson , L. K. Doolittle , M. W. G. Schneider , et al., “Organization of Chromatin by Intrinsic and Regulated Phase Separation,” Cell 179, no. 2 (2019): 470–484.e21, 10.1016/j.cell.2019.08.037. 31543265 PMC6778041

[4] S. Ray , N. Singh , R. Kumar , et al., “α‐Synuclein Aggregation Nucleates Through Liquid‐Liquid Phase Separation,” Nature Chemistry 12, no. 8 (2020): 705–716, 10.1038/s41557-020-0465-9. 32514159

[5] S. Yasuda , H. Tsuchiya , A. Kaiho , et al., “Stress‐ and Ubiquitylation‐Dependent Phase Separation of the Proteasome,” Nature 578, no. 7794 (2020): 296–300, 10.1038/s41586-020-1982-9. 32025036

[6] Q. Zheng , Y. Chen , D. Chen , et al., “Calcium Transients on the ER Surface Trigger Liquid‐Liquid Phase Separation of FIP200 to Specify Autophagosome Initiation Sites,” Cell 185, no. 22 (2022): 4082–4098.e22, 10.1016/j.cell.2022.09.001. 36198318

[7] S. Mehta and J. Zhang , “Liquid‐Liquid Phase Separation Drives Cellular Function and Dysfunction in Cancer,” Nature Reviews. Cancer 22, no. 4 (2022): 239–252, 10.1038/s41568-022-00444-7. 35149762 PMC10036213

[8] P. Yang , C. Mathieu , R. M. Kolaitis , et al., “G3BP1 Is a Tunable Switch That Triggers Phase Separation to Assemble Stress Granules,” Cell 181, no. 2 (2020): 325–345.e28, 10.1016/j.cell.2020.03.046. 32302571 PMC7448383

[9] J. H. Ahn , E. S. Davis , T. A. Daugird , et al., “Phase Separation Drives Aberrant Chromatin Looping and Cancer Development,” Nature 595, no. 7868 (2021): 591–595, 10.1038/s41586-021-03662-5. 34163069 PMC8647409

[10] B. Lu , C. Zou , M. Yang , et al., “Pharmacological Inhibition of Core Regulatory Circuitry Liquid‐Liquid Phase Separation Suppresses Metastasis and Chemoresistance in Osteosarcoma,” Advanced Science 8, no. 20 (2021): e2101895, 10.1002/advs.202101895. 34432948 PMC8529446

[11] B. Lu , R. Qiu , J. Wei , et al., “Phase Separation of Phospho‐HDAC6 Drives Aberrant Chromatin Architecture in Triple‐Negative Breast Cancer,” Nature Cancer 5, no. 11 (2024): 1622–1640, 10.1038/s43018-024-00816-y. 39198689

[12] M. Luo , Z. Huang , X. Yang , et al., “PHLDB2 Mediates Cetuximab Resistance via Interacting With EGFR in Latent Metastasis of Colorectal Cancer,” Cellular and Molecular Gastroenterology and Hepatology 13, no. 4 (2022): 1223–1242, 10.1016/j.jcmgh.2021.12.011. 34952201 PMC8881668

[13] M. J. Xie , H. Yagi , T. Iguchi , et al., “Phldb2 Is Essential for Regulating Hippocampal Dendritic Spine Morphology Through Drebrin in an Adult‐Type Isoform‐Specific Manner,” Neuroscience Research 185 (2022): 1–10, 10.1016/j.neures.2022.09.010. 36162735

[14] M. J. Xie , Y. Ishikawa , H. Yagi , et al., “PIP_3_‐Phldb2 Is Crucial for LTP Regulating Synaptic NMDA and AMPA Receptor Density and PSD95 Turnover,” Scientific Reports 9, no. 1 (2019): 4305, 10.1038/s41598-019-40838-6. 30867511 PMC6416313

[15] L. Song , J. Luo , H. Wang , et al., “ *Legionella pneumophila* Regulates Host Cell Motility by Targeting Phldb2 With a 14‐3‐3ζ‐Dependent Protease Effector,” eLife 11 (2022): e73220, 10.7554/eLife.73220. 35175192 PMC8871388

[16] X. Yang , Z. Wang , S. N. Samovich , et al., “PHLDA2‐Mediated Phosphatidic Acid Peroxidation Triggers a Distinct Ferroptotic Response During Tumor Suppression,” Cell Metabolism 36, no. 4 (2024): 762–777.e9, 10.1016/j.cmet.2024.01.006. 38309267 PMC11209835

[17] H. Wang , L. Wang , Q. Zheng , et al., “Oncometabolite L‐2‐Hydroxyglurate Directly Induces Vasculogenic Mimicry Through PHLDB2 in Renal Cell Carcinoma,” International Journal of Cancer 148, no. 7 (2021): 1743–1755, 10.1002/ijc.33435. 33320958 PMC7986127

[18] D. Ge , Y. Shao , M. Wang , H. Tao , M. Mu , and X. Tao , “RNA‐Seq‐Based Screening in Coal Dust‐Treated Cells Identified *PHLDB2* as a Novel Lung Cancer‐Related Molecular Marker,” BioMed Research International 2021 (2021): 1978434, 10.1155/2021/1978434. 34337001 PMC8314042

[19] W. Kang , J. Zhang , T. Huang , et al., “NOTCH3, a Crucial Target of miR‐491‐5p/miR‐875‐5p, Promotes Gastric Carcinogenesis by Upregulating PHLDB2 Expression and Activating Akt Pathway,” Oncogene 40, no. 9 (2021): 1578–1594, 10.1038/s41388-020-01579-3. 33452458 PMC7932926

[20] G. Chen , T. Zhou , T. Ma , T. Cao , and Z. Yu , “Oncogenic Effect of PHLDB2 Is Associated With Epithelial‐Mesenchymal Transition and E‐Cadherin Regulation in Colorectal Cancer,” Cancer Cell International 19 (2019): 184, 10.1186/s12935-019-0903-1. 31346319 PMC6636018

[21] G. Chen , T. Zhou , Y. Li , Z. Yu , and L. Sun , “p53 Target miR‐29c‐3p Suppresses Colon Cancer Cell Invasion and Migration Through Inhibition of PHLDB2,” Biochemical and Biophysical Research Communications 487, no. 1 (2017): 90–95, 10.1016/j.bbrc.2017.04.023. 28392396

[22] A. M. Daulat , M. S. Wagner , S. Audebert , et al., “The Serine/Threonine Kinase MINK1 Directly Regulates the Function of Promigratory Proteins,” Journal of Cell Science 135, no. 17 (2022): jcs259347, 10.1242/jcs.259347. 35971817

[23] B. C. Lim , S. Matsumoto , H. Yamamoto , et al., “Prickle1 Promotes Focal Adhesion Disassembly in Cooperation With the CLASP‐LL5β Complex in Migrating Cells,” Journal of Cell Science 129, no. 16 (2016): 3115–3129, 10.1242/jcs.185439. 27378169

[24] T. Takabayashi , M. J. Xie , S. Takeuchi , et al., “LL5beta Directs the Translocation of Filamin A and SHIP2 to Sites of Phosphatidylinositol 3,4,5‐Triphosphate (PtdIns(3,4,5)P3) Accumulation, and PtdIns(3,4,5)P3 Localization Is Mutually Modified by Co‐Recruited SHIP2,” Journal of Biological Chemistry 285, no. 21 (2010): 16155–16165, 10.1074/jbc.M109.081901. 20236936 PMC2871484

[25] G. Lansbergen , I. Grigoriev , Y. Mimori‐Kiyosue , et al., “CLASPs Attach Microtubule Plus Ends to the Cell Cortex Through a Complex With LL5beta,” Developmental Cell 11, no. 1 (2006): 21–32, 10.1016/j.devcel.2006.05.012. 16824950

[26] S. J. Stehbens , M. Paszek , H. Pemble , A. Ettinger , S. Gierke , and T. Wittmann , “CLASPs Link Focal‐Adhesion‐Associated Microtubule Capture to Localized Exocytosis and Adhesion Site Turnover,” Nature Cell Biology 16, no. 6 (2014): 561–573, 10.1038/ncb2975. 24859005 PMC4108447

