# Supplementary material for: Phase Separation of PHLDB2 Drives EMT and Tumor Progression in Triple‐Negative Breast Cancer
Source: Cancer Med. 2025 Nov 30;14(23):e71308. doi: 10.1002/cam4.71308 (PMC12665185; doi:10.1002/cam4.71308)
Supplement: Supplementary file 1 — Data S1: cam471308‐sup‐0001‐Figures.docx. [file CAM4-14-e71308-s001.docx]

**Supplementary Material**

**
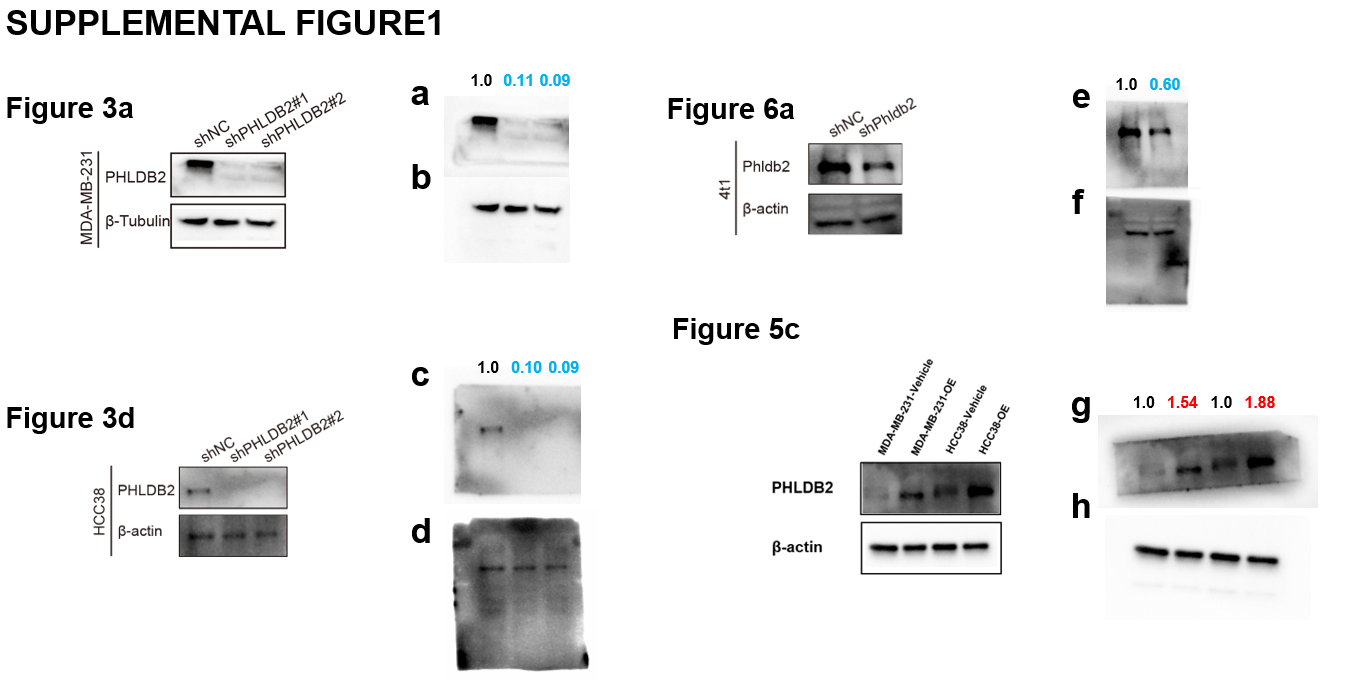
**

**Supplemental Figure 1. Full-length western blot images corresponding to Figures 3a, 3d, 5c, and 6a.**

**a-b**: Western blot analysis showing knockdown efficiency of PHLDB2 in MDA-MB-231 cells using two independent shRNAs (shPHLDB2#1 and shPHLDB2#2), with β-Tubulin as a loading control. Corresponding uncropped blots are shown in panels a (PHLDB2) and b (β-Tubulin).

**c-d**: Western blot validation of PHLDB2 knockdown in HCC38 cells using two independent shRNAs. β-actin was used as the loading control. Full-length blots are shown in panels c (PHLDB2) and d (β-actin).

**e-f**: Western blot confirming reduced Phldb2 expression in 4T1 cells upon shRNA-mediated knockdown. β-actin was used as the loading control. Uncropped blots are provided in panels e (Phldb2) and f (β-actin).

**g-h**: Western blot analysis showing PHLDB2 overexpression in MDA-MB-231 and HCC38 cells compared to vector controls. β-actin serves as a loading control. Panels g and h show the respective full-length blots for PHLDB2 and β-actin.


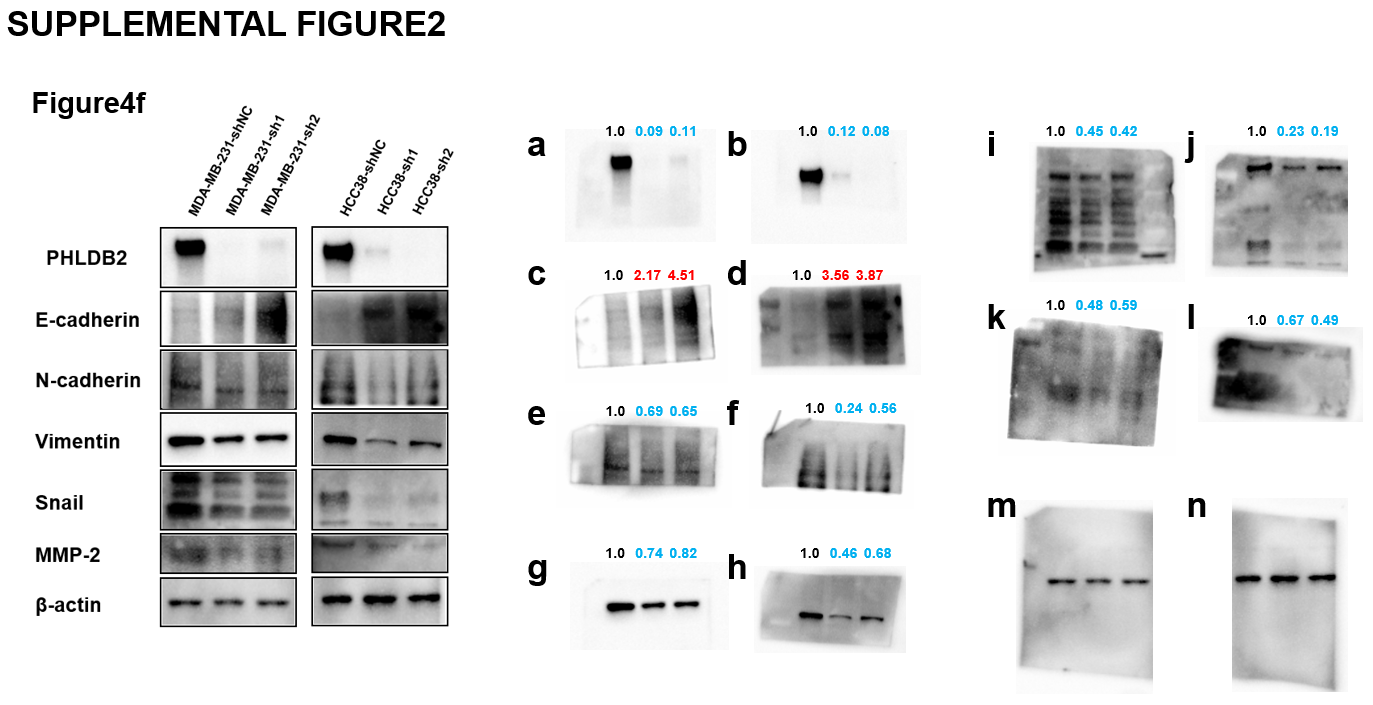


**Supplemental Figure 2. Full-length western blot images corresponding to Figures 4f.**

**a–n**: Western blot analysis showing expression levels of PHLDB2, E-cadherin, N-cadherin, Vimentin, Snail, MMP-2, and β-actin in MDA-MB-231 and HCC38 cell lines following knockdown of PHLDB2 using two independent shRNAs (sh1 and sh2) compared with negative control (shNC). Knockdown of PHLDB2 increases E-cadherin expression while reducing mesenchymal markers (N-cadherin, Vimentin, Snail, and MMP-2), suggesting reversal of the EMT phenotype. Original unprocessed western blot images corresponding to the cropped blots shown in the left panel. Each letter **(a–n)** represents the raw blot for the respective protein in the figure for verification and transparency.
